# Cerebral ischemia, a rare postoperative complication of cervical disc herniation: a case report

**DOI:** 10.11604/pamj.2025.50.67.40590

**Published:** 2025-03-07

**Authors:** Dahmane Elhairech, Mohamed Lmejatti

**Affiliations:** 1 Neurosurgery Department, Hassan II University Hospital, Agadir City, Morocco; 2 Faculty of Medicine and Pharmacy of Agadir, Ibn Zohr University, CF49+F65, Agadir 80000, Morocco; 3 Laboratory of Cellular Biology and Molecular Genetics, Faculty of Sciences, Ibn Zohr University, Agadir, Morocco

**Keywords:** Cervical hernia, ischemic stroke, surgery, medical treatment, case report

## Abstract

Cervical spondylotic myelopathy is a degenerative disease that often requires surgical intervention. Postoperative cerebral ischemia is a rare but serious complication of cervical disc herniation surgery, with its pathophysiology poorly described. Intraoperative carotid manipulation is the most likely cause of this ischemia. We report a case of ischemic stroke as a complication following cervical disc herniation surgery in a 52-year-old patient. The patient developed left hemiplegia and central facial palsy immediately after surgery. Imaging revealed an ischemic stroke in the right Sylvian territory associated with atherosclerotic changes in the right common carotid artery. Early postoperative rehabilitation and antiplatelet therapy led to partial recovery. This case highlights the importance of careful carotid management during surgery and the need for preoperative assessment in high-risk patients.

## Introduction

Cervical disc herniation is a common degenerative disorder often requiring surgical intervention. While surgery is generally successful, it can be complicated by a range of postoperative events. Among these, cerebral ischemia is a rare but serious complication, occurring in only 0.22% of cases. The pathophysiology of postoperative cerebral ischemia following cervical disc herniation surgery is not well understood but is believed to be primarily linked to intraoperative manipulation of the carotid artery. As cervical degenerative disorders increasingly affect a younger population, understanding these risks becomes crucial. This report presents a case of ischemic stroke as a postoperative complication of cervical disc herniation surgery in a 52-year-old patient, aiming to raise awareness of the potential vascular risks associated with such procedures.

## Patient and observation

**Patient information:** a 52-year-old male patient with no significant medical history presented with cervico-brachial neuralgia radiating to the left anterior arm. This symptom persisted for three months and was resistant to medical treatment.

**Clinical findings:** on clinical examination, pyramidal signs were noted in all four limbs, along with intermittent claudication.

**Timeline of current episode:** symptoms of cervico-brachial neuralgia developed over the last three months, and the patient underwent surgery shortly after a diagnosis of cervical disc herniation at C3-C4 and C4-C5.

**Figure 1:**
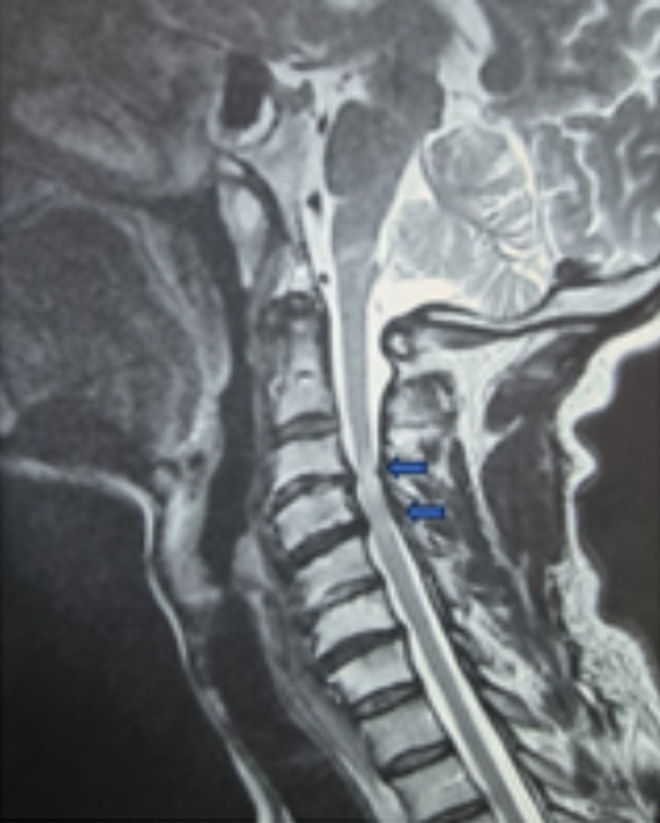
magnetic resonance imaging scan displays two cervical disc herniations at C3-C4 and C4-C5, associated with disco-osteophytic spurs; arrows indicate the location of the disc herniations and thinning of the peri-medullary space

**Figure 2:**
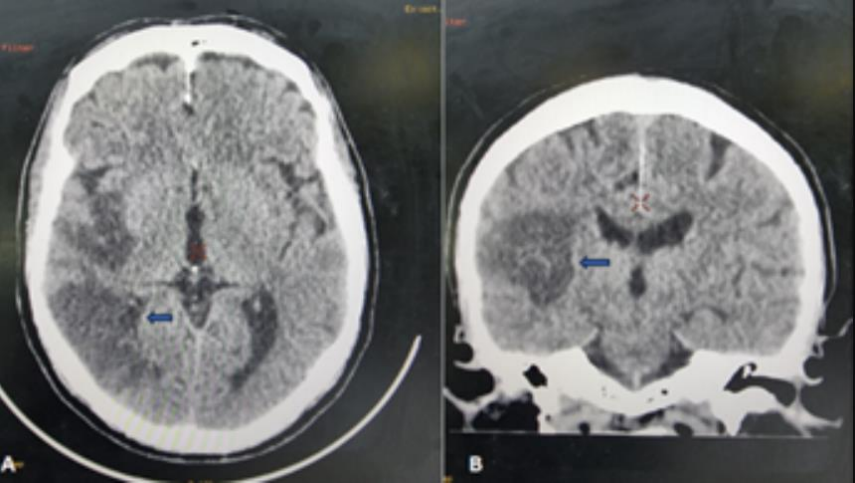
computed tomography scan in axial (A) and coronal (B) sections revealing a right temporo-parietal hypodensity, consistent with ischemic stroke in the Sylvian territory; arrows point to the area of ischemia

**Figure 3:**
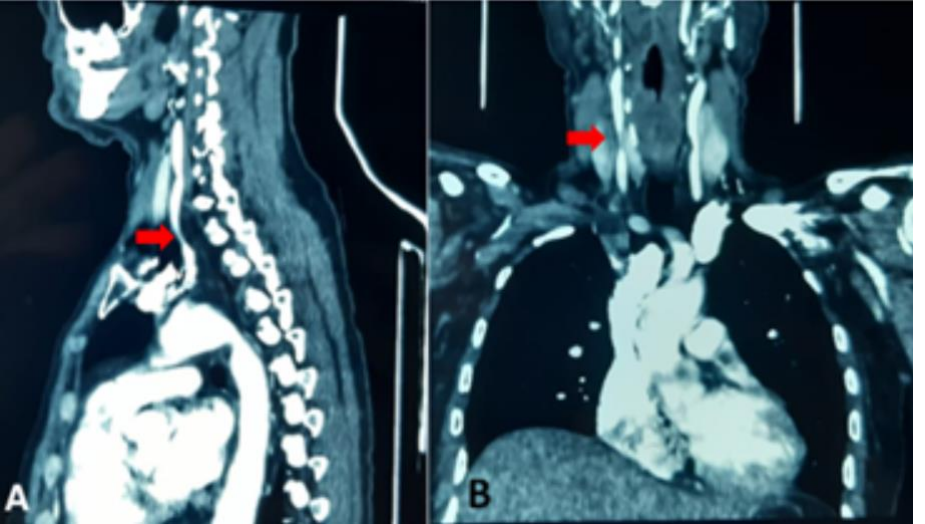
angioscan images in sagittal (A) and coronal (B) views showing an atherosclerotic plaque located in the right common carotid artery; arrows highlight the plaque in the respective sections

**Diagnostic assessment:** cervical Magnetic resonance imaging (MRI) revealed disc herniations at the C3-C4 and C4-C5 levels, with thinning of the anterior peri-medullary space (Figure 1). Postoperatively, a cerebral Computed Tomography (CT) scan without contrast injection in the axial and coronal sections showed right temporo-parietal hypodensity in favor of ischemic stroke in the Sylvian territory (Figure 2 A,B). Angioscan of the neck vessels in the sagittal and coronal sections revealed an atherosclerotic plaque of the right common carotid artery (Figure 3).

**Diagnosis:** ischemic stroke associated with intraoperative carotid manipulation during cervical disc herniation surgery.

**Therapeutic interventions:** the patient underwent anterior discectomy with cervical cage and plate fixation. Postoperatively, the patient developed left hemiplegia and central facial palsy. Antiplatelet therapy was initiated, alongside motor rehabilitation.

**Follow-up and outcome of interventions:** after one year, the patient maintained some degree of left hemiparesis but retained walking autonomy.

**Patient perspective:** the patient expressed relief from his initial symptoms of neuralgia but acknowledged the significant impact of the stroke on his quality of life.

**Informed consent:** it was obtained from the patient and his family for the publication of this case report.

## Discussion

**Epidemiology:** cerebral ischemia following cervical disc herniation surgery is a rare but serious complication. It highlights the potential risks associated with the manipulation of cervical arteries during surgery. Several risk factors may increase the likelihood of ischemic events, including advanced age, a history of atherosclerotic disease, and prior surgical history [1-4]. Although this complication is uncommon, it becomes more significant as the population undergoing cervical disc surgery includes individuals with multiple comorbidities and atherosclerotic changes.

**Diagnosis:** the diagnosis of postoperative cerebral ischemia is typically based on imaging studies, with cerebral computed tomography (CT) being the first-line investigation to identify ischemic changes in the brain. In this case, the brain scan revealed a right temporo-parietal hypodensity, indicating ischemia in the Sylvian territory, which confirmed the diagnosis of ischemic stroke [2,4,5]. Magnetic resonance imaging (MRI) may also be utilized in further evaluation to better assess the extent and location of the ischemic lesion.

**Treatment:** postoperative management for ischemic stroke typically involves antiplatelet therapy or anticoagulation, depending on the timing of the ischemic event. Thrombolytic therapy with tissue plasminogen activators (tPA) may be considered if the stroke occurs within the first few hours of onset, but it is contraindicated in the first 14 days after neurological surgery due to the risk of hemorrhage [4,6]. In this case, the patient was treated with antiplatelet therapy and motor rehabilitation, which significantly contributed to their recovery. The timely initiation of rehabilitation was key to maintaining functional independence.

**Prognosis:** the long-term prognosis for patients who experience cerebral ischemia after cervical disc herniation surgery can vary based on the severity and location of the ischemic lesion. In this case, the patient maintained walking autonomy but continued to experience hemiparesis. Early recognition and intervention, such as the use of antiplatelet therapy and motor rehabilitation, played a crucial role in preventing further neurological deterioration. Despite some residual deficits, the patient was able to regain a level of independence and quality of life after the event [2,6].

## Conclusion

Cerebral ischemia is a rare but significant complication of cervical disc herniation surgery, often caused by intraoperative carotid manipulation. Careful attention to the cervical vascular zone during surgery, with intermittent relaxations during retraction maneuvers, is essential to minimize this risk. Preoperative assessment of the carotid artery, particularly in at-risk patients, should be considered to prevent such complications.
